# Use of in-field bioreactors demonstrate groundwater filtration influences planktonic bacterial community assembly, but not biofilm composition

**DOI:** 10.1371/journal.pone.0194663

**Published:** 2018-03-20

**Authors:** Geoff A. Christensen, JiWon Moon, Allison M. Veach, Jennifer J. Mosher, Ann M. Wymore, Joy D. van Nostrand, Jizhong Zhou, Terry C. Hazen, Adam P. Arkin, Dwayne A. Elias

**Affiliations:** 1 Biosciences Division, Oak Ridge National Laboratory, Oak Ridge, Tennessee, United States of America; 2 Marshall University, Biological Sciences, Huntington, West Virginia, United States of America; 3 University of Oklahoma, Norman, Oklahoma, United States of America; 4 University of Tennessee, Knoxville, Tennessee, United States of America; 5 Lawrence Berkeley National Laboratory, Berkeley, California, United States of America; 6 University of California at Berkeley, Berkeley, California, United States of America; Universita degli Studi di Milano-Bicocca, ITALY

## Abstract

Using in-field bioreactors, we investigated the influence of exogenous microorganisms in groundwater planktonic and biofilm microbial communities as part of the Integrated Field Research Challenge (IFRC). After an acclimation period with source groundwater, bioreactors received either filtered (0.22 μM filter) or unfiltered well groundwater in triplicate and communities were tracked routinely for 23 days after filtration was initiated. To address geochemical influences, the planktonic phase was assayed periodically for protein, organic acids, physico-/geochemical measurements and bacterial community (via 16S rRNA gene sequencing), while biofilms (i.e. microbial growth on sediment coupons) were targeted for bacterial community composition at the completion of the experiment (23 d). Based on Bray-Curtis distance, planktonic bacterial community composition varied temporally and between treatments (filtered, unfiltered bioreactors). Notably, filtration led to an increase in the dominant genus, *Zoogloea* relative abundance over time within the planktonic community, while remaining relatively constant when unfiltered. At day 23, biofilm communities were more taxonomically and phylogenetically diverse and substantially different from planktonic bacterial communities; however, the biofilm bacterial communities were similar regardless of filtration. These results suggest that although planktonic communities were sensitive to groundwater filtration, bacterial biofilm communities were stable and resistant to filtration. Bioreactors are useful tools in addressing questions pertaining to microbial community assembly and succession. These data provide a first step in understanding how an extrinsic factor, such as a groundwater inoculation and flux of microbial colonizers, impact how microbial communities assemble in environmental systems.

## Introduction

Fluctuating geochemical conditions influence aquatic bacterial communities, via their taxon distributions, activity, stability and evolution [1]. Within these communities, microbial activities develop micro-environments that influence pH, the carbon-to-nitrogen ratio, and oxygen levels, but are still subject to the surrounding geochemical conditions. A large proportion of bacterial biomass exists as a complex of cells embedded within an extracellular polymeric matrix composed of DNA, proteins, and polysaccharides [2]. The structure of the biofilm provides considerable advantages compared to those microorganisms in a planktonic mode of growth due to availability of nutritive sources and beneficial interactions within biofilm organisms (e.g., quorum sensing) [3]. However, dispersal and colonization of biofilms or dispersal during biofilm sloughing (later in biofilm development) inherently links biofilm and planktonic microbial communities [4] and thus their differences in active planktonic species may impact subsequent biofilm formation and growth [5].

Previous work has examined the role that contaminants have on groundwater microbial community diversity, structure, function, and biotransformation capabilities [6–18]; but this work has had limited success in linking geochemistry to microbial community structure and overall function. Knowing the interactions between geochemical factors such as pH and chemical species (i.e., Fe^2+^ and Fe^3+^) [19], seasonal change with water content variation [20], mineral composition including relative clay mineral content influencing nutrient mobility [21], headspace atmosphere [22], pore connectivity for flux and percolation [23], and temperature along depth [24], and microbial community response may be promising for better understanding how an environment will respond to an introduced perturbation. Hence, we propose to examine the effect that changing biological and/or geochemical conditions by filtering have on bacterial communities.

In order to elucidate the active bacterial community dynamics altered by biogeochemical factors and discern the difference that geochemistry alone had, we used our recently developed in-field bioreactor system [22]. These bioreactors may be operated continuously to monitor microbial community (i.e. planktonic (mobile) and biofilm (sedimentary)) responses and can be operated at the groundwater source indefinitely. Additionally, the bioreactors are small and versatile, which minimize the sediment, groundwater, and headspace required while maximizing the ability to better discern biogeochemical responses to specific perturbations. Field-scale bioremediation studies often employ large-scale simulations of natural conditions to provide insight on microbial community response to geochemical augmentation [25–27]. For example, at the uranium-contaminated Rifle Aquifer site in Colorado, well injections of acetate were used to stimulate microbial uranium reduction in subsurface-sediment samples [28, 29]; however, these large-scale efforts often have economic and experimental design limitations. The small-scale bioreactor system used in this study, compared to a large-scale field experiment, is more cost-effective, less labor intensive, requires a shorter time between experiments (i.e. minimal/no recover time), and provides continuous monitoring of the microbial community. Hence, the in-field bioreactor system bridges the gap between field and laboratory studies.

In this study, we address specific parameters that may influence bacterial community assembly with filtered or unfiltered groundwater supplied from a subsurface well in the West Bear Creek Valley in Oak Ridge, Tennessee. We aimed to discern the influence that natural groundwater geochemistry and incoming groundwater microbial species had on both planktonic and biofilm bacterial communities as assessed via 16S rRNA gene sequencing. The experiment was designed to measure general factors that affect an established community that form in these bioreactors (i.e. mock community), and not necessarily an exact representation of the site of interest. The geochemistry examined included: pH, oxidative-reductive potential (ORP), conductivity, and dissolved oxygen (DO). Sediment composition and pH were monitored throughout the experiment to confirm that changes were not artificially introduced due to experimental design. We hypothesized that filtration would not influence geochemistry, but due to the exclusion of microbial inocula, result in different bacterial communities within both planktonic and biofilm habitats. Specifically, we propose that the microbial community from the filtered treatment, specifically the planktonic community, are less variable over time as they do not receive an influx of microbial inoculum from the source groundwater well. Bioreactors receiving filtered groundwater simulated the effects of the geochemistry alone (or at least particles <0.2 μm), while the bioreactors receiving unfiltered groundwater acted as controls. This study, which establishes the role of geochemistry on bacterial communities may aid in better predictions for understanding perturbations within groundwater systems which may ultimately be informative for potential groundwater remediation strategies.

## Materials and methods

### Environmental background site

**Fig 1:**
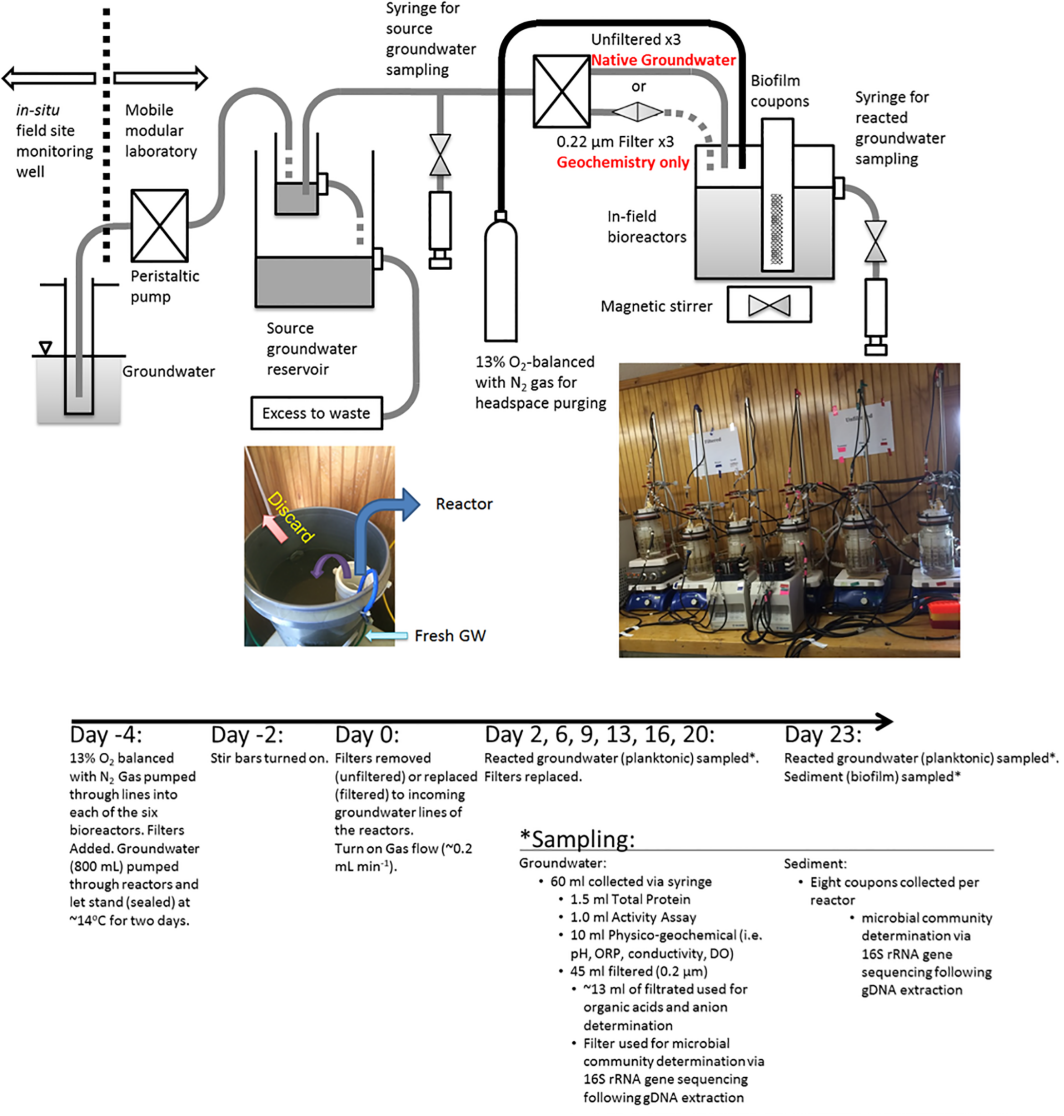
Experimental design. Schematic representation and description of the bioreactor setup and sampling schedule.

The Integrated Field Research Challenge (IFRC) background site is an approximately 1.63 km^2^ field located in West Bear Creek Valley, Oak Ridge, Tennessee [30]. The area is underlain by interbedded shales, siltstones, and limestones of the Conasauga Group that have undergone post-depositional deformation [31]. In-field bioreactors were located within an on-site temperature controlled modular laboratory near (~2 m) the monitoring well, FW305 [22] located at 35.941039,-84.335839. Permission to conduct the study was given by the Y12 National Security Complex. The field studies did not involve endangered or protected species. Detailed information regarding installation, casing, screen, groundwater packer, peristaltic pumps and liquid lines have been previously described [22]. In July of 2015, groundwater was pumped from ~3.7 m below the surface of well FW305 into a sterile, covered 200-mL primary container with an overflow into a larger reservoir in the modular laboratory that was exposed to air momentarily, prior to being pumped directly into each of the reactors (Fig 1). The exposure to air was considered acceptable for the experimental design as the O_2_ levels of the groundwater has been reported to be slightly microaerophilic, yet more reducing than the atmosphere [18].

### Bioreactor experimental set-up and sampling

The detailed preparation of each bioreactor has been reported [22], including assemblage, sterilization, gas purging, groundwater dripping through a tube breaker, and stirring. Each bioreactor had an external water jacket to maintain temperature at the *in-situ* well groundwater temperature, ~14°C. All cooling lines were temperature-stable reinforced flexible lines equipped with quick release fittings. Groundwater was transferred via peristaltic pumps at ~0.22 mL min^-1^ (or about a 2-day turnover rate) using positive gas pressure comprised of 13% oxygen balanced with nitrogen (Airgas, Radnor PA). The gas composition was selected as it represented similar O_2_ levels from the source groundwater, 1.3 mg L^-1^ or ~13% [18]. Each bioreactor contained 800 mL of groundwater and 100 mL of headspace, with eight removable biofilm coupons, as previously described [22].

Three bioreactors were set-up per treatment, with six bioreactors in total being operated simultaneously (Fig 1). Experimental treatments included: 1) incoming groundwater filtered through a 0.22 μm nitrocellulose filter (Merck Millipore, Burlington MA) that was replaced every two days (experimental treatment termed “filtered” hereafter; geochemistry alone influence), and 2) incoming groundwater entered bioreactor without prior filtration (experimental treatment termed “unfiltered” hereafter; geochemistry plus incoming microbial species influence). Initially, each bioreactor was filled with unfiltered groundwater, filters were then added to the inflow lines of all bioreactors to prevent any excess dripping into the system, and bioreactors were acclimated for 48 hr. Bioreactors were incubated for an additional 48 hr with mixing with magnetic stir bars, but no groundwater flow. After this four-day acclimation period, water samples were collected (day 0) and gas flow started. At day 0, 60 mL water was sampled from each bioreactor, filters were removed (unfiltered treatments) or replaced depending (filtered treatments), groundwater flow into all bioreactors was initiated, and the experiment continued for approximately three weeks, ending in August 2015. The length of the experiment was chosen because it was previously reported that significant microbial changes could be observed within a daily-to-weekly time-scale as monitored by these bioreactor systems [22].

Water samples were collected at the outflow port with a sterile syringe twice weekly for approximately 3 weeks (day = 0, 2, 6, 9, 13, 16, 20, and 23). Each time, 60 mL water was collected the following analyses: (1) 1.5 mL for protein analysis via bicinchoninic acid protein assay (BCA^™^, Pierce, Rockford, IL)[32] with bovine serum albumin as the standard, (2) 1 mL for dehydrogenase analysis with 2- (p-Iodophenyl)-3 (p-nitrophenyl)-5-phenyl tetrazolium chloride (INT) to assess microbial activity [33], (3) ~10 mL for water physicochemical properties of incoming and reacted groundwater (i.e. pH, oxidation-reduction potential (ORP), conductivity, and dissolved oxygen (DO), (4) ~45 mL passed through a sterile 0.2 μm nitrocellulose filter (Merck Millipore) in an EMD Millipore Swinnex^™^ filter holder (Thermo Fisher Scientific, Waltham, MA) and stored at -80°C for gDNA extractions. Lastly, the filtrate was collected and 13 mL was dispersed into a 15-mL falcon tube and stored at 4°C until analyzed for organic acids and anions via ion chromatography. At the end of the experiment (day 23) eight coupons from each bioreactor were collected and stored at -80°C for gDNA extractions.

### Biogeochemical characterization biofilm sediments, of source groundwater, and of bioreactor water

Each coupon had 2 g (wet wt.) of homogenized freezer-milled sediment (Freezer/Mill^®^ 6870, SPEX^®^ Sample Prep, Metuchen, NJ), which was obtained during construction of the source well and stored at -80°C. The physical characteristics reflecting the mineral composition of the sediment used for establishing biofilm communities was characterized to determine if the core sediment had X-ray diffraction (XRD)-detectable alterations before and after the autoclaving and freezer-milling processes and at the conclusion of the experiment. The mineral composition of the pulverized sediment was characterized with an X-ray diffractometer (X’pert PRO, PANalytical, Natick, MA) equipped with Mo-Kα radiation at 55 kV/40 mA between 5‒35° 2θ with 1.5° 2θ/min [34]. For XRD analyses, representative mixed solid samples from each of the experimental types were selected. Each coupon sample was washed three times with DI-water prior to XRD analysis. For the semi-quantification of mineral constituents, the reference intensity ratio method was used [35]. Sediment pH was measured with core sediment reacted with 10 mM CaCl_2_ solution (sediment:solution ratio of 1:2 kg L^-1^) equilibrated for 60 minutes on an end-to-end shaker. Testing these parameters was important to confirm that experimental design did not transform the samples, and potentially influence the growth of an irrelevant microbial community. This was significant since modified physical properties of the sediment could impact indigenous microbial community due to a change of their environmental niche such as solid phase change through oxidation of thermal transformations that develop to sparingly reducible phases.

The pH, ORP, conductivity, and DO of the source and reacted groundwater were measured with an electrochemistry benchtop meter, Orion^™^ Versa Star Pro^™^ (Thermo Fisher Scientific), combined with Ross^™^ Ultra pH/ATC Triode (Orion^™^ 8157 BNUMD), Triode ORP probe (Orion^™^ 9179 BNMD), conductivity cell (Orion^™^ 013005MD), and DO probe (Orion^™^ 083005MD), respectively. The bicarbonate concentration was measured by titrating filtered groundwater stored in the refrigerator to pH 4.5 with 10 mM HCl (Dosimat 765, Metrohm, Switzerland). When sampling volume was less than 10 mL, the sample was diluted with deionized water (>18MΩ) to allow the probe to be submerged.

Ferrous iron, Fe(II), was used an indicator for the process of microbial metal-reduction from source and reacted groundwater samples. Fe(II) was measured with 1,10 phenanthroline by colorimetric detection (Ferrous Iron Reagent Powder Pillows, HACH, Loveland CO) (UV-spectrometer, Hewlett Packard 8453); the detection limit was ~0.05 mg L^-1^ Fe^2+^.

The organic acids (lactate, acetate, propionate, formate, succinate, fumarate, and citrate) and anions (chloride, bromide, nitrate, sulfate, and phosphate) were assayed at room temperature with a Dionex^™^ ICS-5000 DP Dual Pump, Dual Column system (Thermo Fisher Scientific) equipped with an AS11-HC column with a KOH gradient of 1–60 mM as reported elsewhere [22]. 5 mL volumes of each sample were injected with an auto-sampling system, Dionex^™^ AS-DV, and stored for no more than 48 hr at room temperature prior to injection.

### DNA extraction, Illumina MiSeq sequencing, and associated statistical analyses

For bioreactor fluid samples (referred to as “planktonic” hereafter), gDNA was extracted from filters following a freeze-grinding method [36] and quantified with PicoGreen^®^ (Quant-iT^™^ PicoGreen^®^ dsDNA assay kit; Thermo Fisher Scientific). For each coupon (N = 8 per bioreactor, referred to “biofilms” hereafter) samples were taken out of the -80°C storage, thawed on ice, and gDNA was isolated from the ~2-g (wet wt.) coupon sediment by following previously described methods [37, 38]. After gDNA was extracted, each sample was passed through the UltraClean^®^ Microbial DNA isolation kit (MoBio, Carlsbad, CA), starting with MD2 solution addition step. Final nucleic acid concentrations and purity were measured with a Nanodrop^™^ ND-1000 (Thermo Fisher Scientific).

Planktonic and biofilm prokaryotic communities were determined by 16S rRNA SSU gene sequencing on an Illumina^®^ Miseq^™^ system (Illumina, San Diego, CA), following previous protocols [22, 39] with sample libraries prepared with Illumina^®^ MiSeq^™^ Reagent Kit v3 (2 x 300 cycles). The sequencing data were processed in QIIME (version 1.9.1) [40]. The raw sequence reads were multiplexed and quality filtered (Phred quality threshold of Q20), then chimeric sequences were identified and removed with QIIME implemented USEARCH algorithm [41, 42]. Then sequences were clustered in operational taxonomic units (OTUs) at a 97% sequence similarity with the open reference workflow and the greengenes reference database (version 13.8) (http://genegenes.lbl.gov) [43]. OTUs were classified with taxonomic affinities with the Naïve Bayesion RDP Classifier [44] with a bootstrap confidence threshold of 80%. Contaminants (unclassified sequences at the domain level, chloroplasts, and mitochondria) and rare OTUs (sequence count <10) were filtered and data was rarefied at 2,300 sequences per sample to minimize the loss of samples. The final, rarefied bacterial community dataset had 3,670 OTUs and 181,700 sequences. Shannon diversity, Faith’s phylogenetic diversity (PD), and Good’s coverage was calculated via QIIME with the rarefied dataset. Sequencing data (joined fastq files) are archived at the Sequence Read Archive (SRA) under the BioProject Accession PRJNA414405.

A 2-way ANOVA model (function *aov* in stats package) was used to determine if microbial diversity changed over time and between filtered and unfiltered samples for planktonic communities. If a significant effect was detected for a time, a Tukey’s HSD test was used for multiple, pairwise significance comparisons. Further, a 2-way ANOVA model was performed to assess if diversity differed within biofilm versus planktonic communities (referred to as “habitat”) and between filtered and unfiltered samples at the end of the experiment (day 23). The top twenty most abundant OTUs, which comprised ~38% of all sequences combined, were similarly analyzed. Several of these abundant OTUs were designated the same classification– 7 taxonomic identities were found across these 20 dominants, therefore all OTUs with the same taxonomy had abundance summed. Prior to analysis, dominant OTU relative abundance was log_10_-transformed to meet assumptions of normality.

Bray-Curtis dissimilarity matrices were calculated separately for planktonic communities only to discern time and treatment (filtered versus unfiltered) effects and for day 23 planktonic and biofilm communities to discern treatment and habitat effects. Dissimilarity matrices were then input into non-metric dimensional scaling (NMDS) ordinations for visualization of community composition across our experimental design (function *metaMDS* in vegan; [45]). The number of dimensions was set to 2 (k = 2; [45]) and number of random starts to 20 which provided acceptable stress values indicating NMDS ordinations are representing variation in community composition appropriately. A permutational multivariate ANOVA was performed (permutations = 999) to partition how much variation is explained by either time, treatment, or habitat (planktonic vs biofilm)[46].

## Results

### Geological characteristics of sampled sediment cores

The sediment used for the coupons in the bioreactors were examined for mineral composition. Sediment pH (pH 4.5) did not change throughout the experiment and was similar to previously reported values from a site located nearby (pH 4.3 ± 0.02) [30]. As expected, the XRD-pattern remained the same before and after the autoclaving and freezer-milling processing (S1 Fig). Minimal peak differences (i.e. peak intensity and overall shape) were observed among samples, and were like the pre-experiment samples with two exceptions. First, the relative intensity of the illite peak was increased for the samples assayed after gDNA extraction (~5–10° 2θ range). Secondly, the background signal or noise of the instrument output was less for the post gDNA extraction samples. A new X-ray tube was installed between pre/post processing (S1a and S1b Fig) and post gDNA extraction (S1c and S1d Fig) samples, which may alone account for some of the variability observed.

### Physicochemical properties of groundwater and reacted water

The source groundwater pH remained at pH 7.36–7.55 for the length of the experiment, following an initial reading at 6.85 (S2a Fig). Minimal differences (i.e. overlapping error bars across all treatments) were observed between the filtered and unfiltered reacted groundwater. However, groundwater pH steadily increased from pH 7.72 ± 0.03 to 8.31 ± 0.14 for filtered and from 7.73 ± 0.02 to 8.41 ± 0.09 for unfiltered and both declined to pH 7.86 ± 0.06 and pH 7.88 ± 0.03, respectively, at the last time point, day 23. The increased reacted groundwater pH when compared to source groundwater pH is likely due to continuous stirring, which removes headspace gas including biogenic CO_2_ resulting in steady bicarbonate concentration (~175 mg L^-1^) under microbial activity and elevated pH levels [22], discussed later (i.e. hydrogenase activity and total protein levels).

After an initial 2-day equilibration, the ORP was consistent between the source and reacted groundwater increasing from ~50 mV at day 0 to >250 mV by day 6, and remaining between 275–400 mV until the termination of the experiment (S2b Fig). Slightly higher ORP of source groundwater may have been due to the air exposure during the residence in the primary container. Conductivity among all samples was also similar, equilibrating at 279 μS cm^-1^ for source groundwater, 268 ± 2.0 μS cm^-1^ for filtered, and 264 ± 0.6 μS cm^-1^ for unfiltered reacted groundwater on day 9 (S2c Fig), and the lower EC of reacted groundwater (i.e. standard deviations smaller than the symbol size) regardless of filtering than the source groundwater was sustained throughout the experiment. As conductivity equilibrated early on, this suggests that identifiable mineral composition changes are unlikely to have occurred throughout the process. Slight drops in conductivity of reacted groundwater for filtered (220–280 μS cm^-1^) and unfiltered (208–280 μS cm^-1^) compared to source groundwater (252–291 μS cm^-1^) is likely to originate from either a simple adsorption of dissolved elemental species to the surfaces within the bioreactor or microbial precipitation dissolved species into relatively sparingly soluble solid phase under the shifted pH-Eh condition. All DO values (3.80–4.43 mg L^-1^) on day 0 maintained at 3.76–4.40 mg L^-1^ for filtered and 3.50–4.51 mg L^-1^ for unfiltered from day 2 to day 20 except the last day, day 23. These increased values in DO, when compared to the source groundwater, are likely due to the supplied gas (13% O_2_-balanced N_2_) being slightly more oxygenic, in addition to the brief exposure of the groundwater to atmospheric air prior to entering the bioreactor, and are therefore an artifact of experimental design [22]. However, DO from continuously pumped groundwater measured 2.24–2.62 mg L^-1^ until day 23. (S2d Fig). These results are similar to a previous study [22] with DO measurements of at 0.8–1.3 mg L^-1^ and 4.9–8.8 mg L^-1^ for source and reacted groundwater, respectively.

Filtered and unfiltered reacted groundwater exhibited minor variation of chloride (Cl^-^) at 1.3–1.9 mg L^-1^ and 1.3–2.0 mg L^-1^, nitrate (NO_3_^-^) at 0.4–1.4 mg L^-1^ and 0.3–1.4 mg L^-1^, and sulfate (SO_4_^2-^) at 4.7–5.5 mg L^-1^ and 4.6–5.6 mg L^-1^, respectively (S3 Fig). Fluoride (F^-^) was below detection for all samples. Bicarbonate was also examined (S3 Fig) and ranged from 165–233 mg L^-1^ filtered and 154–187 mg L^-1^ for unfiltered, corresponding to silicate rock as the source [47]. Lactate spiked at day 9, more in the unfiltered (7.3 μM) than the filtered (2.0 μM), followed by transient acetate spikes for both unfiltered and filtered, 11.3 μM and 9.3 μM, respectively (S4 Fig). Formate (0.4–3.8 μM) showed less variation than acetate (0.8–5.0 μM) throughout the experiment regardless of treatment (S4 Fig). For all samples Fe(II) measured at or below the detection limit (<0.05 mg L^-1^).

### Microbial activity, diversity and community composition

**Fig 2:**
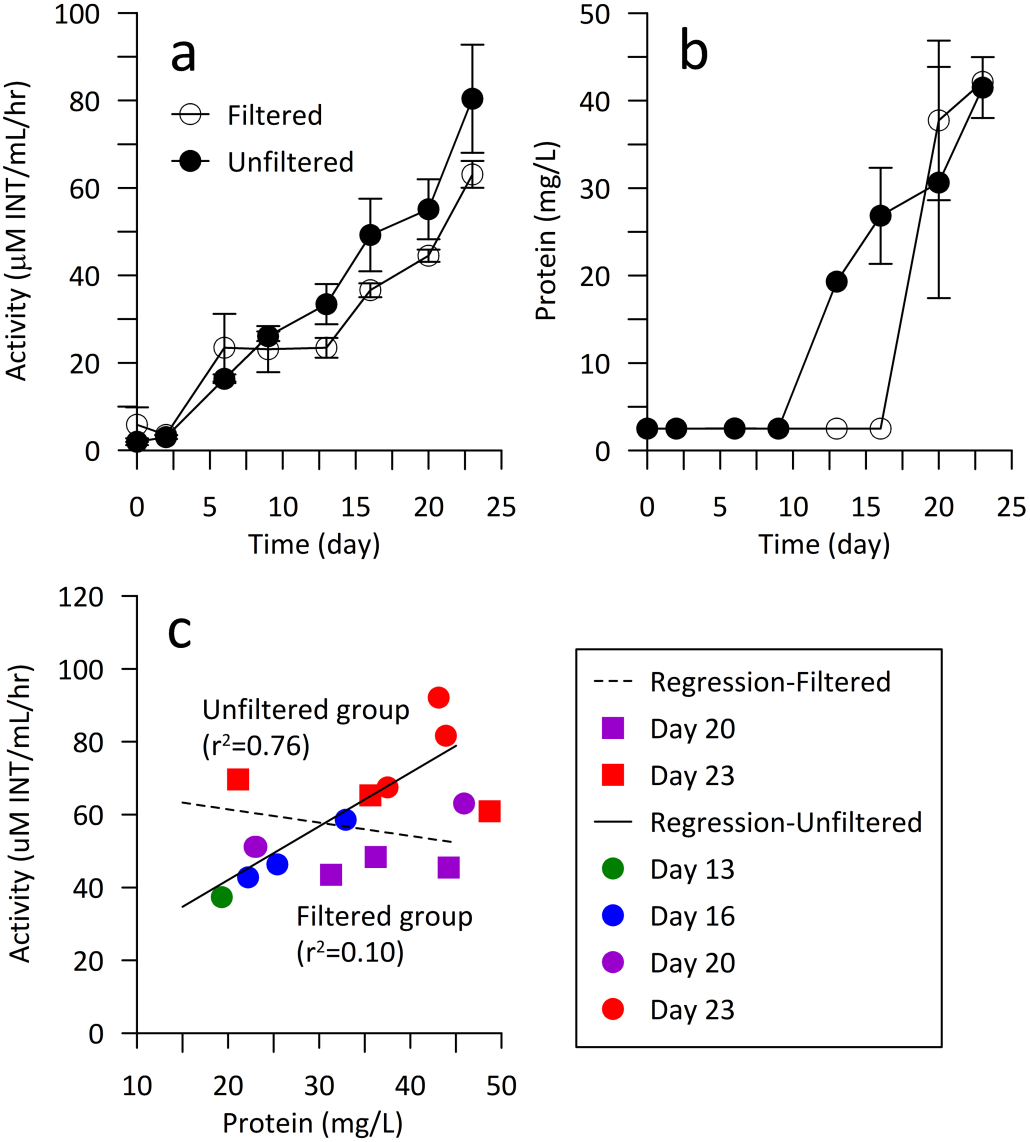
Microbial activity within the reacted groundwater. (a) Dehydrogenase activity and (b) total protein (± standard deviation) of planktonic communities (i.e. reacted groundwater) in bioreactors from filtered (open square) and unfiltered (closed square) groups. (c) Variation in hydrogenase activity versus total protein.

Multiple analyses were conducted to measure microbial activity within the reacted groundwater community, including a dehydrogenase activity assay, and total protein concentration (Fig 2). Similar trends were observed for reacted groundwater from unfiltered and filtered treatments (Fig 2a). The reactors supplied by unfiltered groundwater exhibited slightly higher dehydrogenase activity (2.0 ± 0.8 to 80.4 ± 12.4 μM INT mL^-1^ hr^-1^) than those of filtered groundwater (3.6 ± 0.144 to 65.3 ± 4.4 μM INT mL^-1^ hr^-1^). Both groups increased steadily over time; however, based on protein concentration, the unfiltered group had shorter lag time (Fig 2b); the unfiltered reacted groundwater samples had detectable protein at day 13 (19.3 mg L^-1^), while the filtered groups had detectable protein at day 20 (37.2 ± 6.5 mg L^-1^), one week later. The temporal ratios of activity and protein of the microbial communities in the planktonic phase were examined (Fig 2c). Unfiltered samples appeared to increase in activity with increasing protein concentrations from days 13 to 23 (R^2^ = 0.73, p = 0.001). The filtered samples were determined to be not significant (p = 0.54), i.e. no trend was observed between activity and protein levels.

Unfiltered, planktonic microbial communities were greater in Shannon diversity (5.1 ± 0.2; F_1,44_ = 5.24, p = 0.03) and phylogenetic diversity, PD, (23.7 ± 1.0; F_1,44_ = 57.0, p < 0.001) than filtered (Shannon: 4.6 ± 0.1, PD: 17.7 ± 0.7). Furthermore, PD differed over time regardless of treatment (F_7,38_ = 9.15, p < 0.001). Specifically, day 0 and day 2 communities were lower in PD compared to most other time points (Tukey’s HSD, p < 0.05). At day 23, biofilms had greater Shannon diversity (6.2 ± 0.1) and PD (26.1± 1.2) than planktonic communities (Shannon: 4.8 ± 0.4, PD: 24.5 ± 2.3; F_1,11_ = 10.0, p = 0.01), but neither differed between filtered and unfiltered treatments (p ≥ 0.13).

**Fig 3:**
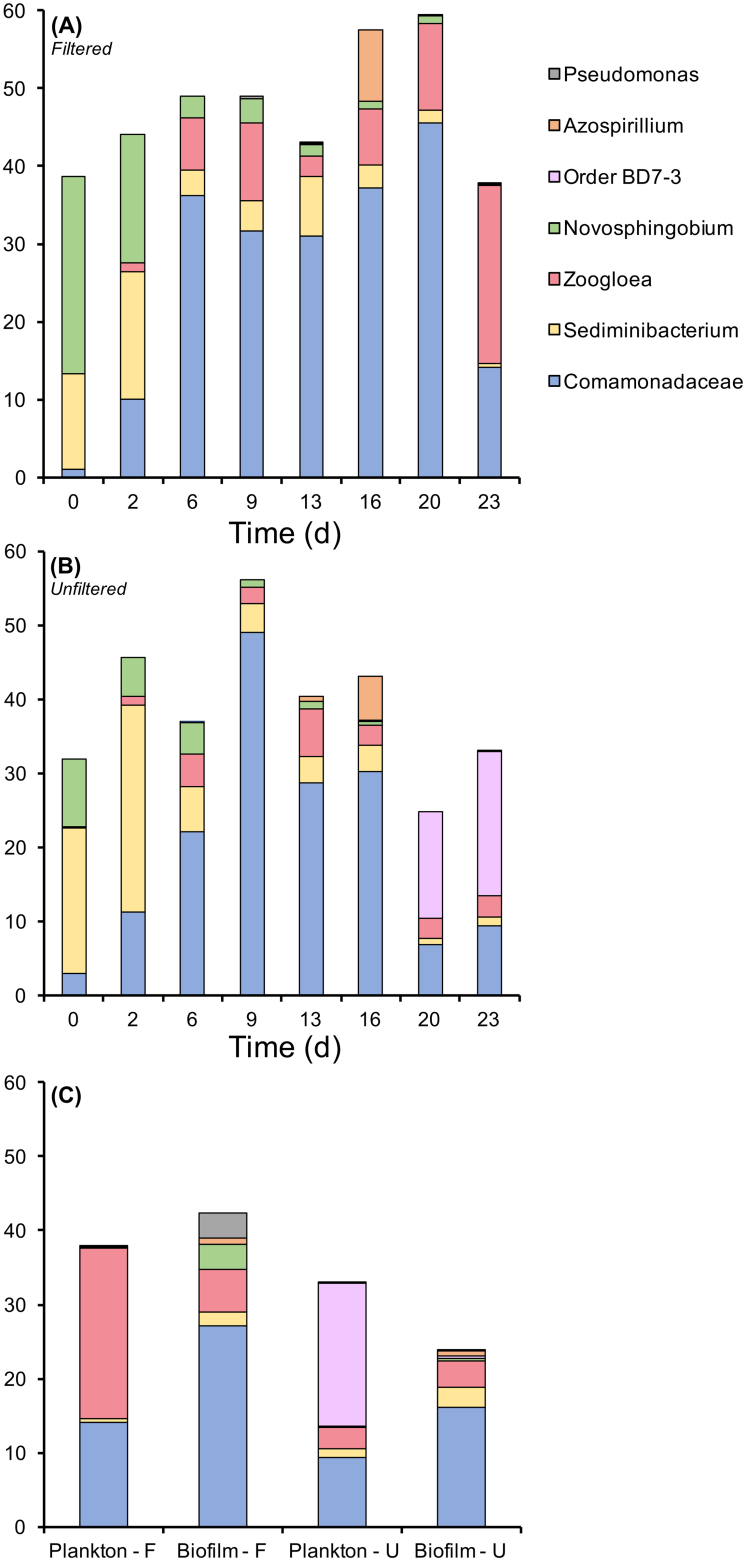
Most abundant bacterial OTUs. The top 20 most abundant OTUs, combined into the same taxon if classified similarly, for planktonic communities in filtered (Panel A) and unfiltered treatments (Panel B) over time, and within plankton and biofilm communities at the end of the experiment (day 23; Panel C).

**Fig 4:**
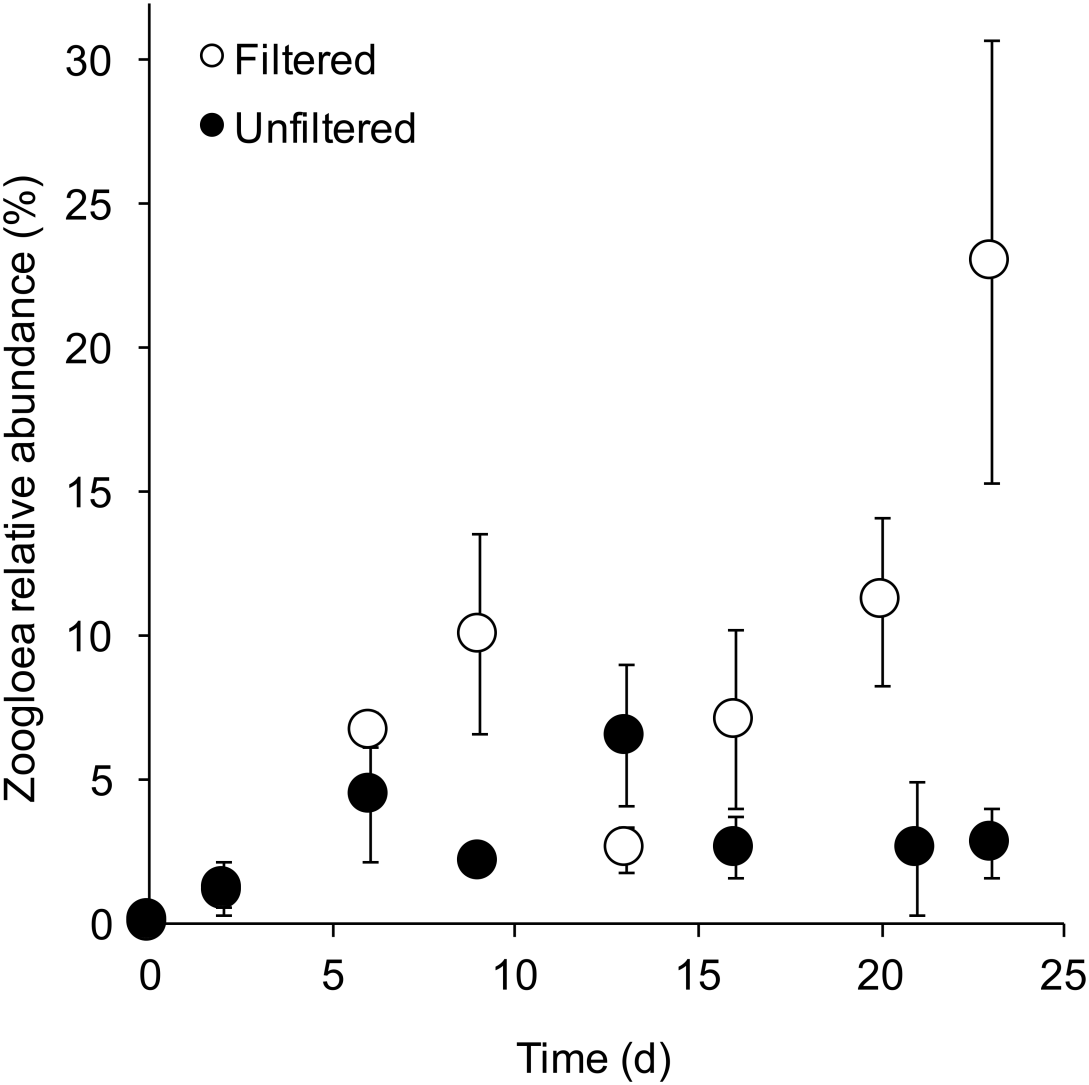
The genus *Zoogloea* relative abundance over time. Planktonic *Zoogloea spp*. mean relative abundance (± standard error bars) over time within filtered and unfiltered bioreactors. *Zoogloea* abundance differed over time and between filtered and unfiltered bioreactors. Notably, *Zoogloea* had a time x filtered/unfiltered interaction—its abundance increased over time in filtered bioreactors and remained similar over time in unfiltered bioreactors.

The dominant bacterial phyla (> 1% relative abundance) for all samples were primarily comprised of Proteobacteria—Betaproteobacteria (53.8% sequences), Alphaproteobacteria (20.3%), Gammaproteobacteria (6.3%), and Deltaproteobacteria (2.4%). In addition to these Protoebacterial, Bacteroidetes (11.2%) and Actinobacteria (1.8%) were also abundant. The most abundant OTUs were classified to the Betaproteobacterial family *Comamonadaceae* and the genus *Zoogloea*, the Bacteroidetes genus *Sediminibacterium*, Alphaproteobacterial order BD7-3 and genus *Novosphingobium* and *Azospirillum*, and lastly, the Gammaproteobacterial genus *Pseudomonas*. Out of these 7 dominant bacterial groups (identified from the 20 most abundant OTUs), two varied over time and/or between filtered and unfiltered bioreactors (Fig 3). The Alphaproteobacterial genus, *Novosphingobium*, declined in abundance over time (F_7,37_ = 89.44, p < 0.001), and was, on average, greater in filtered planktonic communities compared to unfiltered (F_1,44_ = 9.0, p = 0.005). The Betaproteobacteria *Zoogloea*, a common freshwater microbe, had a significant time and treatment interaction (F_7,37_ = 5.62, p = 0.02). *Zoogloea* increased in abundance in filtered bioreactors over time, but remained the same abundance, on average, in unfiltered bioreactors (Fig 4). At day 23, three groups differed between habitats (Fig 3). *Comamonadaceae* (F_1,11_ = 5.65, p = 0.04), *Sediminibacterium* (F_1,11_ = 6.93, p = 0.03), and *Novosphingobium* (F_1,11_ = 9.91, p = 0.01) were greater in biofilms compared to planktonic. Lastly, *Zoogloea* did not differ between habitats (p = 0.32), but was greater, on average, in filtered treatments compared to unfiltered regardless of habitat (F_1,11_ = 7.36, p = 0.02).

**Fig 5:**
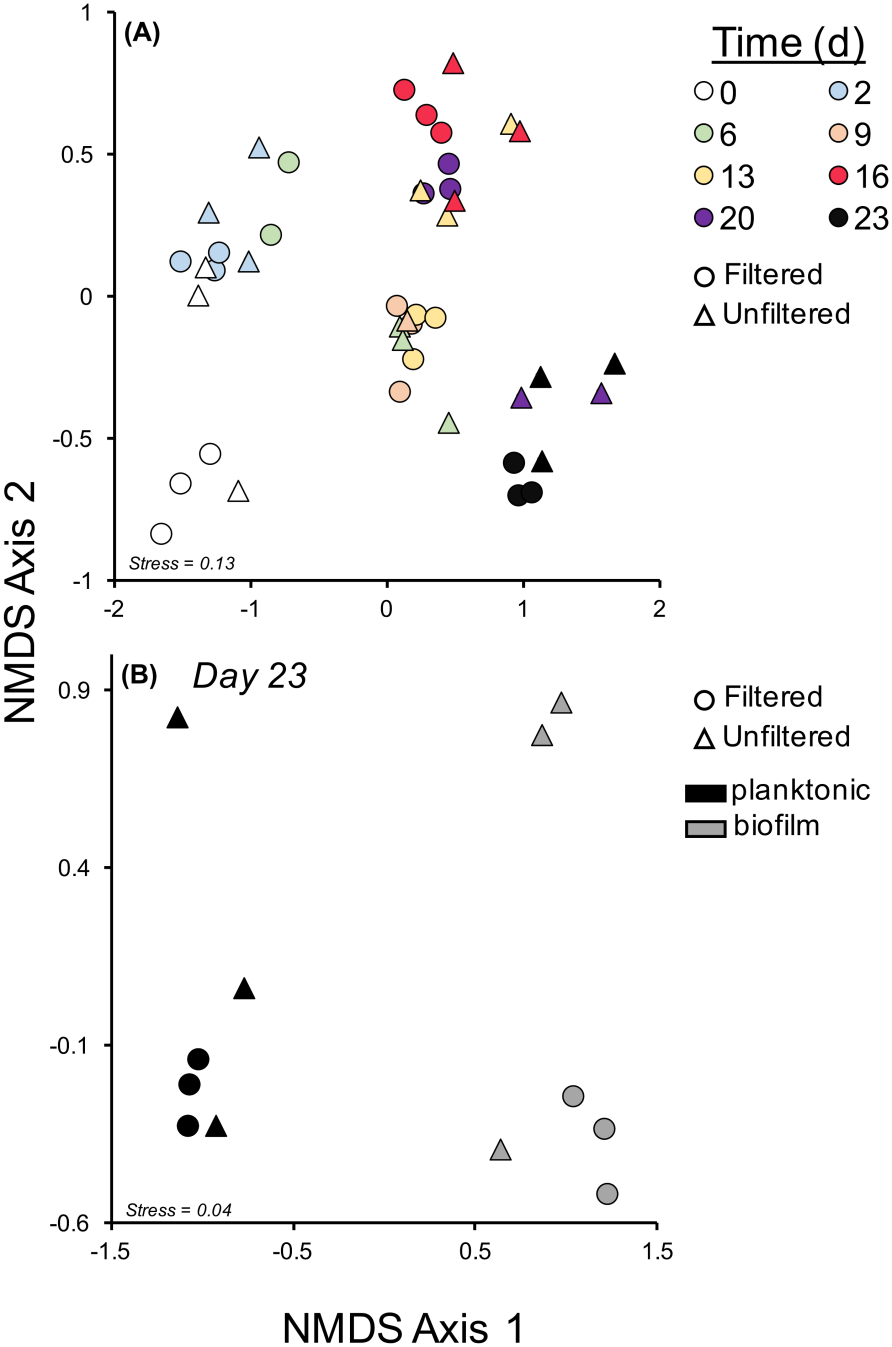
Bacterial community dynamics over time. Non-metric Multidimensional scaling (NMDS) ordinations for bacterial planktonic communities over time for filtered (circles) and unfiltered (triangles) samples (Panel A) and for biofilm (gray symbols) and planktonic communities (black symbols) at the end of the experiment (day 23; Panel B).

Based on Bray-Curtis distance, variation in planktonic community composition was explained by time (perMANOVA: p = 0.001), filtered/unfiltered treatment (p = 0.008), and their interaction (p = 0.001). Most of the variation was related to time (R^2^ = 0.46), with the interaction term (R^2^ = 0.17) and main effect of filtered/unfiltered (R^2^ = 0.03) treatment secondarily influential (Fig 5a). On day 23, both habitat (planktonic versus biofilm; p = 0.001) and the interaction between habitat and filtration (p = 0.05) significantly explained variation in bacterial community composition, with habitat (R^2^ = 0.34) being the most influential (filtration R^2^ = 0.12). The main effect of filtration did not have a strong influence (R^2^ = 0.12, p = 0.06; Fig 5b).

## Discussion

Understanding microbial community assembly and stability in response to a disturbance, such as fluctuations in geochemistry or colonization of exogenous microbial inoculum, is essential for linking microbial function to community structure. Here, we consider the role of filtering the incoming groundwater and its impact on bacterial community stability in planktonic and biofilm communities. Established microbial communities can be resistant (non-responsive) or resilient to environmental conditions, with the latter being detected by a change in a microbial property initially but steadily returning to a pre-disturbance state [48, 49]. In this study, we found planktonic bacterial communities were sensitive to incoming groundwater due to large differences in dominant bacteria taxa and overall community composition when filtration occurred. Due to the limited differences in geochemistry measured, it is likely that these results are due to incoming microbial inoculum in unfiltered bioreactors, thus indicating the importance of microbe-microbe interactions during community assembly [50]. Also, biofilm bacterial communities, although similar between our experimental treatments, were significantly different from their corresponding planktonic communities. These findings would suggest that, regardless of filtration, bacteria taxa capable of biofilm colonization and succession are driven by other factors not accounted for in this study [51].

### Impact of filtration on groundwater geochemistry within the reactors

The groundwater from well FW305 has been reported to be nutrient poor in terms of organic acids [22], including μM concentrations of lactate, acetate, and formate, which was consistent with measurements taken in this study. The increase of lactate, most significantly in the unfiltered (7.3 μM) versus filtered (2.0 μM), was likely caused by breakdown of trace amounts of influx carbon sources that transiently spiked after day 9 coinciding with Cl^-^ and NO_3_^-^ peaks, followed by transient acetate spikes at days 13 through 16 for both the filtered and unfiltered treatments. The reason for this is not certain, because the precipitation in total (~25 mm) occurred on days 1 and 2 with trace amounts on day 8, but rainwater percolation might not be the main reason due to the shallow groundwater.

Bicarbonate initially spiked at day 2 for filtered (223 ± 36 mg L^-1^) and unfiltered (187 ± 43 mg L^-1^), followed by constant measurements at 170 mg L^-1^ for both treatments, 165–174 mg L^-1^ fir filtered and 162–177 mg L^-1^ for unfiltered, respectively. It was unclear why these spikes, along with large standard deviation, of bicarbonate for the early time points were so high. This might arise from large sample dilution required (up to 200-fold) for proper measurements initially, which did not exceed five-fold dilution later. Furthermore, the constant purging conditions with 13% O_2_-balanced with N_2_ likely facilitated removal of microbial respiration of CO_2_ and maintained similar geochemical condition regardless of filtration.

The reason for greater DO levels within the bioreactors compared to the groundwater source is unclear, and has been observed while using these bioreactors previously [22], but may also be a combination of exposure to 13% oxygen while groundwater reacted for 48 hr (Dilution Factor = 0.021/hr) and air permeation into bioreactors through the output port compared to short exposure of source groundwater to air at the primary container even though gas resistant Viton tubing was used at all connections. This increased DO may have led to some oxic stress on the microbial community.

For all samples Fe(II) measured at or below the detection limit and is likely due to Fe(OH)_3_ being the most stable iron phase in aqueous solution at pH 7‒8 and Eh 200‒400 mV [52]. Furthermore, since the reacted groundwater pH was >pH 8 after day 2, Fe(III)-reduction was unlikely to be favorable to occur. A trend of increased reacted groundwater pH relative to the source groundwater pH has been previously reported under similar conditions [17]. Further, nitrate- [53], sulfate- [54] and Fe(III)- [55] reduction consumes protons and may have contributed to the observed increase in pH, regardless of filtration. Together, the incoming groundwater had very similar anionic chemistry and no major anions seemed to participate in the mineral-water interaction and potential microbial reduction due to the oxic headspace. Overall, there was no difference observed in the anions examined between the treatments, i.e. filtration. The reasoning for the measured minor peak of nitrate observed on day 8 is likely due to an influx or plume of nitrate from the surrounding groundwater source (S3 Fig).

### Microbial community responses to filtration

Following the acclimation period, on day 0, the microbial planktonic communities regardless of treatment were comprised mainly of *Novosphingobium* (~10‒30%) and *Sediminibacterium* (~10‒20%), consistent with previous reports of groundwater from this site [18, 22]. Members of the genus *Novosphingobium* belong to the Alphaproteobacteria, are obligate aerobes, and are best known for their ability to degrade a wide variety of aromatic hydrocarbons (e.g. benzene, toluene, and phenol) [56] found at this site [18]. Members of the genus *Sediminibacterium* belonging to the phylum Bacteroidetes, are aerobic, and have only recently been identified and have been found in sediment [57], freshwater [58, 59], and soil [60]. Proteobacteria were found to be the dominant phylum of bacterial life from this experiment and agrees with prior experiments that used groundwater from this site [22], and have been reported previously to be a common dominant phylum in freshwater sources (i.e. stream biofilms)[61], marine environments [62, 63], and soil [62]. Additionally, throughout the time-course of this experiment, temporal turnover occurred not only in the overall OTU community composition, but also with the dominant taxa, which differed between filtered and unfiltered treatments. However, at the completion of the experiment, the biofilm bacterial community are relatively similar regardless of treatment, suggesting that incoming microorganisms did not significantly alter the biofilm bacterial community once it had established. This observation suggests that bacteria in a biofilm lifestyle may be more influenced by deterministic processes [64]. Recent work demonstrates that deterministic drivers are influential for microbial biofilm communities, yet these drivers may differ for planktonic communities, thus leading to relatively greater influence of stochasticity in planktonic community [65]. Previous work utilizing the bioreactor system, and examining oxygen as a perturbation on microbial community dynamics, had shown observable changes in composition within days to weeks [22], thus our temporal sampling represents a realistic timeframe for understanding how filtration may impact groundwater microbial communities.

Biofilms were more diverse than planktonic communities, consistent with previous findings [66]; however, but were unaffected by filtration similar to biofilm microbial community composition. From this we infer that the biofilm bacterial community is more stable than the planktonic, at least short term (days to weeks), albeit for these communities, this may represent a mid-to even late successional stage.

Significant rainfall events occurred on day 2 and day 16, which likely led to the observed microbial community response on day 6 and day 20, respectively, in unfiltered bioreactors. Prior to the first rainfall event, *Novosphingobium* and *Sediminibacterium* were the dominant taxa, yet after this event, the Betaproteobacteria group *Comamonadaceae* became dominant, potentially due to dispersal of this group from precipitation infiltration. A similar event occurred after the rainfall event at day 16 –*Comamonadaceae* was replaced by the Alphaproteobacterial order BD7-3 as the dominant taxon due either to rainfall event dispersal of this group or the concurrent environmental changes accompanying this event. For the unfiltered samples, following the rainfall event on day 16, the Alphaproteobacterial order BD7-3 encompassed ~15% of the total OTUs by day 20 and was not detected in the filtered samples throughout the experiment. We presume, therefore, that microorganisms of this order were inhibited by filtration into the bioreactors. Furthermore, order BD7-3 was not significant within the biofilm community at the end of the experiment. There is limited information regarding order BD7-3 [67], although they have been identified in activated sludge [68, 69], and contaminated soils [70], including their role in hydrocarbon degradation. Overall, the bacterial communities were sensitive to both the incoming groundwater and particles greater than 0.2 μm, consistent with previous findings [48, 49].

Most significantly, throughout the experiment, *Zoogloea* increased in relative abundance in the filtered samples (from <1% to >20%) but not for the unfiltered samples (~2–5%) and decreased (to about 2%) in relative abundance following both rain events. *Zoogloea* produce a gelatinous matrix that encompass the bacteria into a floc that floats to the surface allowing the bacteria to be continuously exposed to air [71] and has been considered ubiquitous bacteria that form sludge flocs in sewage treatment plants [72–74]. As the bioreactor is designed to have an air/liquid interface this result is not surprising. However, filtration allowed *Zoogloea* to become a dominant microbial taxon over time within these bioreactors under tested conditions. Because geochemical properties are consistent between treatments (filtered vs unfiltered), we reason that filtration may have excluded *Zoogloea* competitors, allowing *Zoogloae* to increase in abundance. Furthermore, *Zoogloea* utilizes a diversity of organic carbon sources for growth [71] that are readily available in these systems. Therefore, in the absence of introduced microbial competitors, such as the case in the filtered samples, it is likely that *Zooglaea* can proliferate. Furthermore, the filtration process may have impacted other factors that may also contribute to these observations.

## Conclusion

From this experiment, we conclude that excluding particles >0.2 μm affects in-field bioreactor microbial communities, not only in overall OTU frequencies, which included many rare OTUs likely not heavily influential in microbial activity, but also for dominant microbial taxa which may be important for linking structure to function. Additionally, flux of microbial cells after rain events were a major driver for fluctuations observed in the unfiltered planktonic samples resulting in a less stable microbial community in these bioreactors. *Comamonadaceae* appeared to be a dominant taxon over time regardless of treatment, whereas order BD7-3 was only able to proliferate in the unfiltered planktonic samples likely due to its exclusion during filtration. Lastly, *Zoogloea* was the only taxon which displayed different patterns of relative abundance between treatments thus suggesting the importance of exogenous microbial taxa for defining the prevalence of this genus. This study provides a first step at understanding the relative importance of microbial inocula in groundwater microbial community assembly across both water column and biofilm specific habitats. Additional studies are required to better understand specific mechanisms regulating microbial community dynamics observed such as shifts in electron acceptor/donors, influence of specific exogenous microbial inocula, and further characterization of groundwater geochemistry.

## Supporting information

S1 Fig X-ray diffraction analysis of sediment coupon samples. The XRD patterns of representative samples from in-field bioreactors at IFRC site, Oak Ridge, Tennessee. Samples are from a) initial sediment acquired, b) sediment post autoclaved and freezer-milled processes, c) reacted sediment from filtered reactors after nucleic acid extraction, and d) reacted sediment from unfiltered reactors after nucleic acid extraction. Abbreviation: Ab, albite; Ill, illite; Kao, kaolinite; Qz, quartz.
(TIF)

S2 Fig Source and reacted groundwater physical properties. Physical properties of source (dashed line) and reacted groundwater in bioreactors of filtered (open square) and unfiltered (closed square) groups.
(TIF)

S3 Fig Source and reacted groundwater anionic properties. Anionic properties of reacted groundwater in bioreactors of filtered (open square) and unfiltered (closed square) groups.
(TIF)

S4 Fig Reacted groundwater organic acid concentrations. Variation of organic acids of reacted groundwater in bioreactors of filtered (open square) and unfiltered (open square) groups.
(TIF)
