# Supplementary figures and images for: Use of in-field bioreactors demonstrate groundwater filtration influences planktonic bacterial community assembly, but not biofilm composition
Source: PLoS One. 2018 Mar 20;13(3):e0194663. doi: 10.1371/journal.pone.0194663 (PMC5860781; doi:10.1371/journal.pone.0194663)

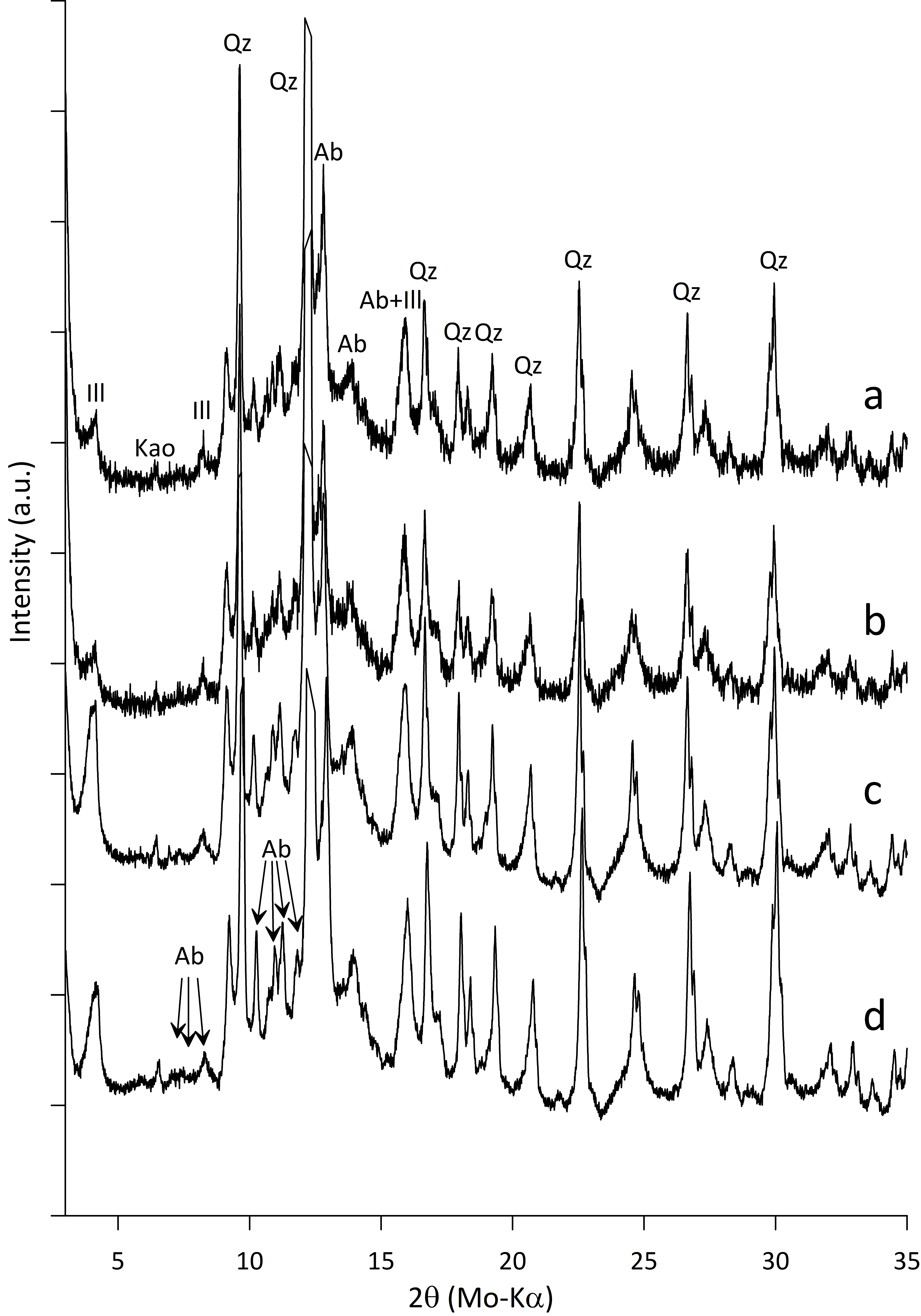

Supplement: S1 Fig — The XRD patterns of representative samples from in-field bioreactors at IFRC site, Oak Ridge, Tennessee. Samples are from a) initial sediment acquired, b) sediment post autoclaved and freezer-milled processes, c) reacted sediment from filtered reactors after nucleic acid extraction, and d) reacted sediment from unfiltered reactors after nucleic acid extraction. Abbreviation: Ab, albite; Ill, illite; Kao, kaolinite; Qz, quartz. (TIF) [file pone.0194663.s001.tif]

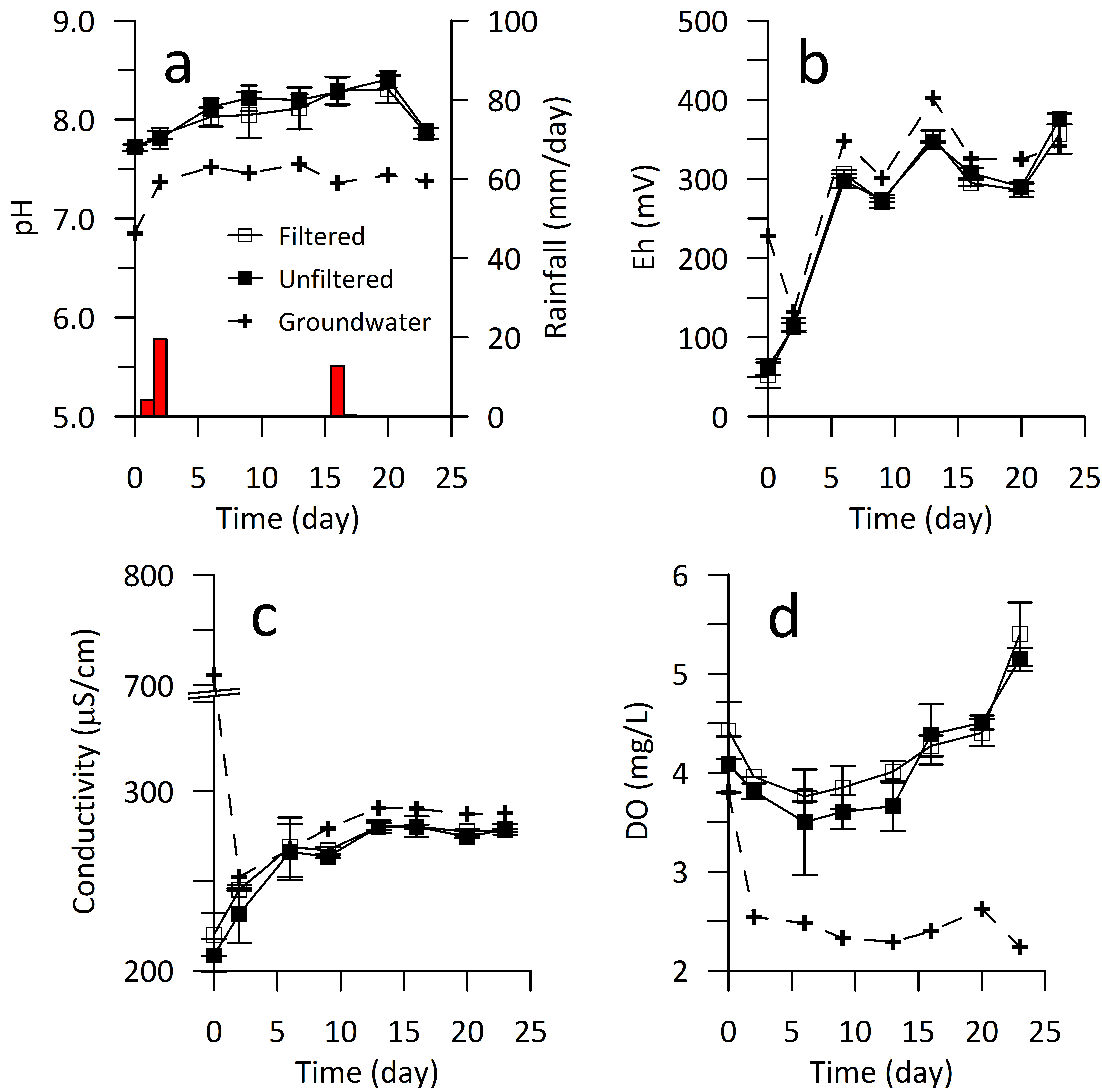

Supplement: S2 Fig — Physical properties of source (dashed line) and reacted groundwater in bioreactors of filtered (open square) and unfiltered (closed square) groups. (TIF) [file pone.0194663.s002.tif]

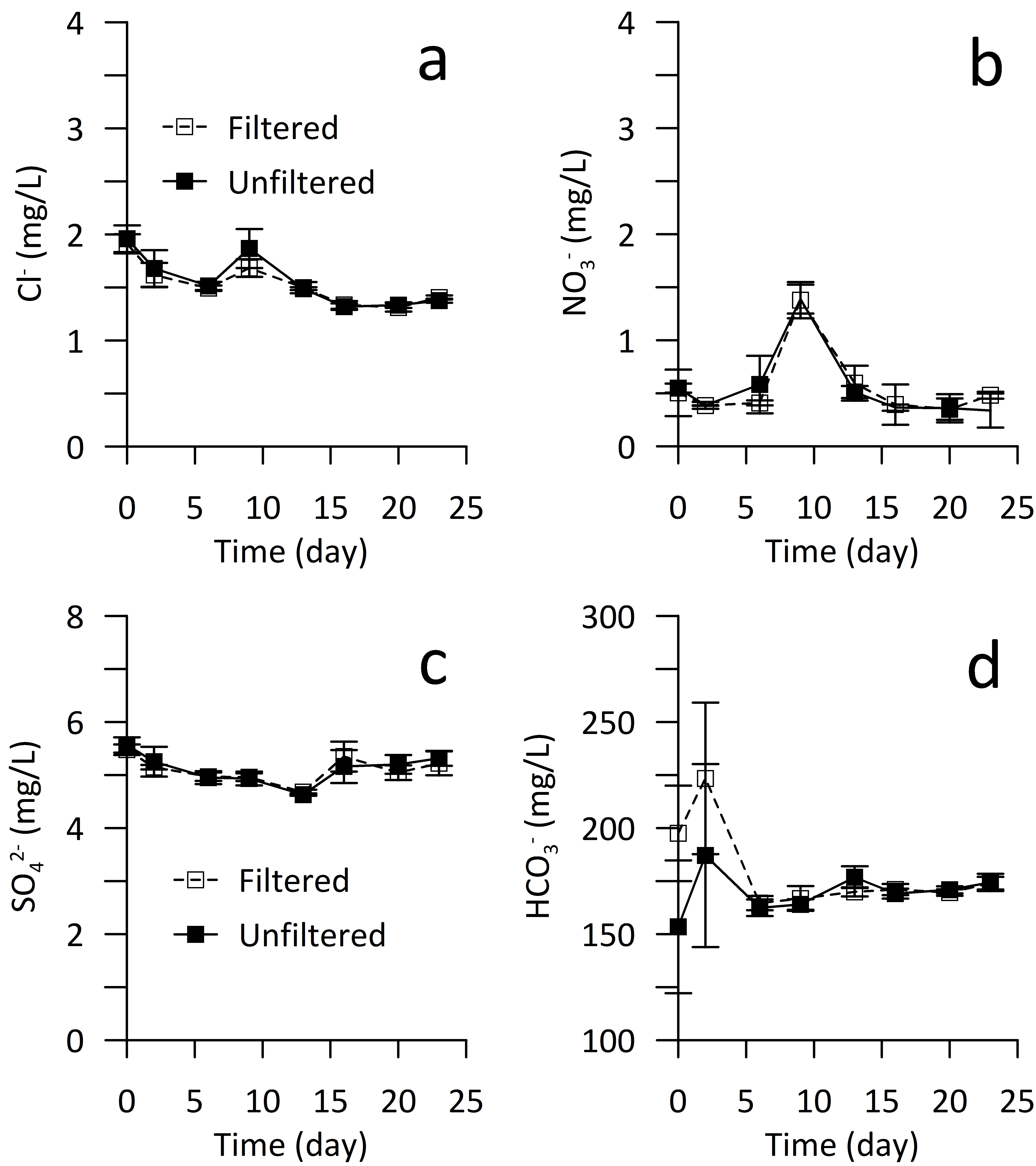

Supplement: S3 Fig — Anionic properties of reacted groundwater in bioreactors of filtered (open square) and unfiltered (closed square) groups. (TIF) [file pone.0194663.s003.tif]

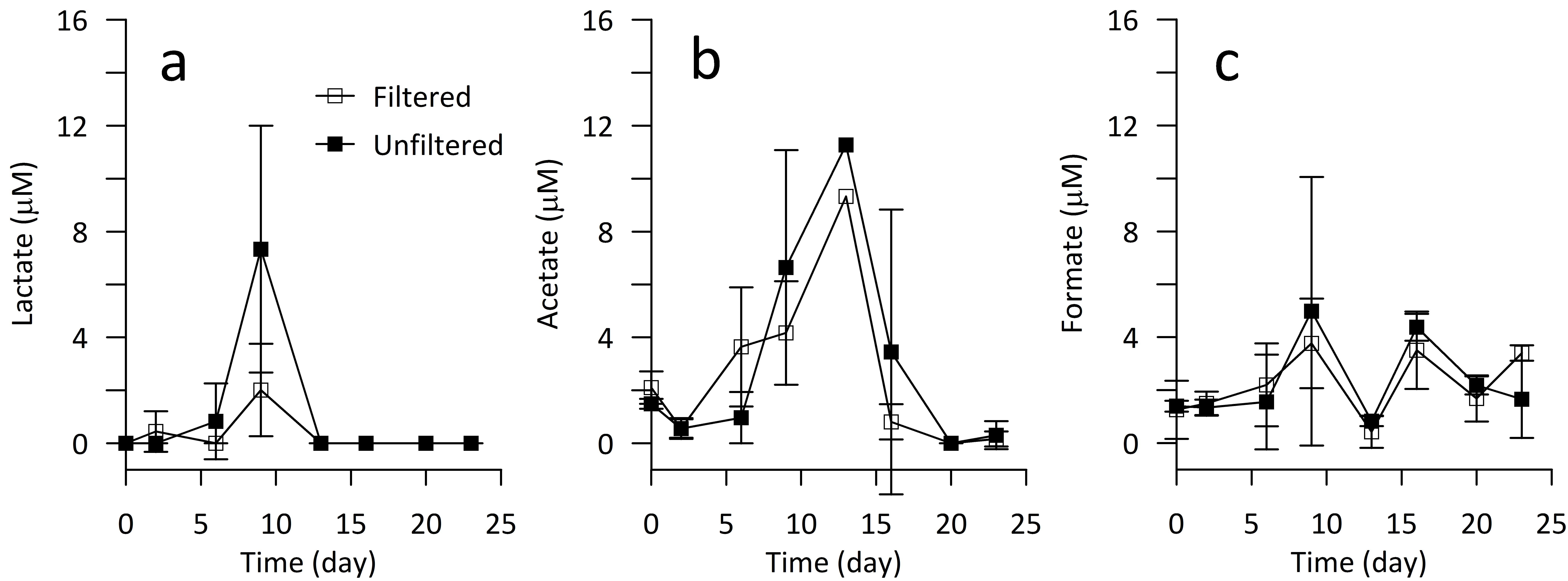

Supplement: S4 Fig — Variation of organic acids of reacted groundwater in bioreactors of filtered (open square) and unfiltered (open square) groups. (TIF) [file pone.0194663.s004.tif]
